# Safety and Efficacy of Chyawanprash as a Prophylaxis Treatment for COVID-19: A Systematic Review and Meta-Analysis of Randomized Control Trials

**DOI:** 10.7759/cureus.71532

**Published:** 2024-10-15

**Authors:** Shubham Sharma, Gayathree Sethuraman, Khushboo Kumari

**Affiliations:** 1 Public Health, Indian Institute of Public Health, Gandhinagar, IND; 2 Public Health, Triyog Ayurved Hospitals, Chennai, IND; 3 Public Health, Indian Council of Medical Research, Rajendra Memorial Research Institute of Medical Sciences, Patna, IND

**Keywords:** chyawanprash, complementary alternative medicine, covid-19, hcru, srma

## Abstract

Amidst the multifaceted challenges posed by COVID-19, interest in complementary and alternative medicine (CAM) has risen. Chyawanprash (CP), an Ayurvedic formulation, is recognized for its multifunctional properties. This systematic review aims to evaluate its safety and effectiveness as a COVID-19 prophylactic. A literature search was conducted for articles published within the past three years from databases such as Cochrane Library, PubMed, and Google Scholar. A total of 1008 articles were identified, and 34 were included for full-text screening, resulting in five randomized controlled trials (RCTs) meeting the eligibility criteria. Meta-analysis was performed using a random effects model, with results represented in odds ratio (95% confidence intervals (CIs)), and publication bias presented through funnel plots. The five clinical trials conducted in India analyzed 153,343 participants. In the meta-analysis, the odds ratios for COVID-19-positive patients, COVID-19-positive in symptomatic patients, healthcare resource utilization (HCRU), and adverse events were 0.45 (95% CI 0.06-3.08, p = 0.41), 0.37 (95% CI 0.01-12.86, p = 0.58), 0.95 (95% CI 0.18-5.04, p = 0.95), and 0.94 (95% CI 0.63-1.40, p = 0.75) respectively. Notably, significant heterogeneity was observed among studies including both COVID-19-positive cases (I^2 ^= 80%, p = 0.007) and symptomatic COVID-19 cases (I^2 ^= 83%, p = 0.01), whereas non-significant zero heterogeneity was observed in HCRU (I^2 ^= 0%, p = 0.74) and adverse events (I^2 ^= 0%, p = 1.00). The meta-analysis reveals a trend in the efficacy of CP as prophylaxis for COVID-19 treatments. However, owing to high heterogeneity and bias, further research is recommended.

## Introduction and background

COVID-19, a multifaceted pandemic characterized by soaring mortality rates, long-term health impact, the specter of vaccine escape, and the emergence of novel variants, has resulted in an exceptional public health crisis for the global community [1-3]. In tandem, the impact of COVID-19 on biomarkers and its strain on healthcare resources underscore the importance of innovative and holistic approaches to combat the pandemic [4,5].

Amidst the labyrinth of challenges, the resurgence of interest in complementary and alternative medicine (CAM) offers a ray of hope. Traditional remedies, deeply rooted in centuries-old healing traditions, have garnered attention for their potential to bolster immunity and mitigate disease risk [6]. Among these, Chyawanprash (CP), an ancient Ayurvedic formulation, has emerged as a subject of particular interest [7,8]. Renowned for its diverse blend of herbs (cinnamon or *Cinnamomum verum*, clove or *Syzygium aromaticum*, and cardamom or *Elettaria cardamomum*), spices, fruits (amla or Indian gooseberry, ashwangandha or Indian ginseng, etc), and honey, Chyawanprash is acclaimed for its purported immunomodulatory properties, improved health outcomes, comparative (compared to standard care) reduced healthcare resource utilization (HCRU), and its ability to promote holistic well-being and improved quality of life (QoL) [8-11].

As conventional pharmaceutical interventions confront challenges such as vaccine efficacy and the threat of emerging variants [3], integrating traditional medicines into mainstream healthcare assumes newfound significance [12,13]. However, amidst the promising potential of CAM, a critical need exists for rigorous scientific evaluation to ascertain safety, efficacy, and potential interactions with conventional treatments [14,15].

In this context, the current systematic review and meta-analysis (SRMA) seeks to shed light on the safety and efficacy of CP as a prophylactic measure against COVID-19. By synthesizing evidence from randomized controlled trials (RCTs) and examining its impact on biomarkers, HCRU, and QoL, we aim to provide a comprehensive understanding of CP's role as prophylaxis in the pandemic.

## Review

Methodology

The review was conducted according to the Preferred Reporting Items for Systematic Reviews and Meta-Analyses (PRISMA) guidelines and was registered in the International Prospective Register of systematic reviews (PROSPERO) with the registration number CRD42023403106.

Study eligibility criteria

As per the PRISMA guidelines, we followed a systematic search for peer-reviewed RCTs published in the last three years (January 2020 to February 2023) in the following databases: Cochrane, Google Scholar, and PubMed. Additionally, a citation search was also conducted. A comprehensive detail of PICOT (population, intervention, comparator, outcomes, and time) is reported in Table 1. Briefly, we sought to identify studies reporting the usage of CP as a solitary or in combination with other therapies for healthy and/or clinically ill adults (≥18 years) of either sexes, excluding pregnant and lactating women, and children below five years of age, to determine the efficacy and safety of CP in stabilizing biomarkers, and improving HCRU and QoL outcomes.

**Table 1:** PICOT *AYUSH: Ayurveda, Yoga and Naturopathy, Unani, Siddha and Homeopathy. HCRU, healthcare resource utilization; RCT, randomized controlled trial; SRMA, systematic review and meta-analysis; PICOT, population, intervention, comparator, outcomes, and time.

Description	Inclusion	Exclusion
1. Population	Healthy, COVID-19 (affected/cured), vaccinated against COVID-19, children (5 to 17 years), adults (≥18 years), and either gender, participants with co-morbid conditions (either COVID-19 positive or negative)	Pregnant and lactating women, children below five years of age, pre-clinical trial/animal study
2. Intervention	Chyawanprash (with or without milk, and other natural ingredients/medication)	Except what’s in the inclusion criteria
3. Comparator	Allopathy drugs, COVID-19 vaccine, AYUSH*, natural ingredients, standard of care, milk. Any or none	Except what’s in the inclusion criteria
3. Outcome	Inflammation biomarkers, incidence of COVID-19, recovery days from COVID-19, quality of life, serious adverse events, adverse events, HCRU (no. of days in hospital, ventilation use, cost, etc), clinical outcome assessment, severity and recovery days from COVID-19, number of days required for testing negative for COVID-19	Except what’s in the inclusion criteria
4. Study design	RCTs (pilot, phase (I, II, and III)), at least two-arm trial	Observational study, SRMA, single-arm RCT, quasi RCT, qualitative study, pre-clinical trial, review article, pooled analysis, other language, letter to editor, and non-peer-reviewed articles
5. Timeline and language	January 2020 to February 2023. English language only	Except what’s in the inclusion criteria

The search strategy included three MeSH terms: Chyawanprash OR chyavanprash OR ratnaprash; the search strategy differed based on the requirements of the respective electronic database. The search strategy for Cochrane, Google Scholar, and PubMed is reported in Supplementary Table 4.

Screening and analysis

We prepared screening and extraction grids in MS Excel with access only to the authors; we piloted the sheet with two studies and gradually finalized the sheet after approval from the authors. Articles were screened in three levels: L1 included title/abstract (ti/ab) screening, at L2 full-text articles were screened, and data extraction was conducted at the L3 level. Screening of articles obtained from databases was conducted independently by Kk and Gs based on inclusion-exclusion criteria reported in Table 1. A third author Ss resolved the discrepancy at both levels (L1 and L2) if any. Additional/missing information was obtained through e-mail from the corresponding author or principal investigator of the study, as required.

The description of included studies was reported in a narrative and analytical manner, consisting of country, intervention and comparator description, setting of the trial, sponsor, etc. The outcomes were reported qualitatively or in the form of mean, standard deviation, proportion, etc. The basic characteristics and detailed findings of the included studies are reported in Tables 2, 3 [10,16-19], respectively.

**Table 2:** Descriptive reporting of included studies NR: not reported; RT PCR: reverse transcriptase polymerase chain reaction; HCW: healthcare worker. All the trials were conducted in India. *Preparation of Ayush Kwatha: Decoction by boiling 3 g of Kwath churna which contains 04 parts of leaves of Tulsi (*Ocimum sanctum *Linn), 02 parts of Dalchini (bark of *Cinnamomum zeylanicum* Bryn), 02 parts of Shunthi (dried rhizome of *Zingiber officinale* Rosc), and 01 part of Kali Mirch (fruit of *Piper nigrum* Linn) in 150 mL water and reduced to 75 mL. ^Samshamani vati: An Ayurvedic herbal formulation prepared by using a single herb called Guduchi (*Tinospora cordifolia*/Giloy). The coarse powder of Guduchi is boiled until reduced to a semi-solid mass. This is rolled in the form of pills. qam: once daily in the morning; qd: once daily; bid: twice a day; pc: post-dinner/post-cibum.

First author, year	Randomization type	NCT number	Inclusion criteria	Exclusion criteria	Intervention	Comparator	Duration of study (days)	Descriptive outcomes	Sponsor
Mata et al., 2022 [16]	Open-label, multicenter, cluster randomized community-based trial. Colony was the randomization unit, 10 areas for each participating institute within a radius of 5 km for randomization. Out of 10 areas (colonies), one serves as control and others as intervention for willing participants	CTRI/2020/08/027316	Healthy individuals of either sex, aged between 18 and 70 years, residing in the identified Scheduled Caste-dominated area/colony/village	COVID-19 positive, pregnant/planning for pregnancy and lactating women, individuals having any co-morbidities, with the immune-compromised state as in HIV, hepatitis, tuberculosis, and malignancy, and taking corticosteroids or any immunosuppressive therapy. Individuals participating in any other clinical study or any other study one month before the screening and history of hypersensitivity to the study interventions	• Ayuraksha Kit • Chyawanprash: 6 g qam, qd on empty stomach • Ayush Kwatha*: 75 ml qd pc • Samshamani Vati^: 2 tablets (250 mg each) bid • Anu Taila: 2 drops each nostril • Standard preventive measures/guidelines for COVID-19	• Standard preventive measures/guidelines for COVID-19	45	The intervention group had higher proportion of COVID-19-negative cases (98.9%) compared to control group (98.1%) after adjusting for contact history with COVID-19-positive or symptomatic patients. Among the COVID-19-positive cases, symptomatic cases were more in the intervention group, but the incidence of hospitalization (9.8%) was less than the control group (12.5%)	Central Council for Research in Ayurvedic Sciences (CCRAS), Ministry of Ayush, Govt. of India
Godatwar et al., 2021 [10]	Randomized, controlled, prospective, multicentric community-based trial	CTRI/2020/05/024981	Healthy male or female subjects in the age group of 5 years to 70 years (both inclusive)	Pregnant, lactating, confirmed or recovered from COVID-19. Patients with diabetes mellitus; any medical or surgical condition requiring immediate medical or surgical intervention; an immune-compromised status due to human immunodeficiency virus (HIV), hepatitis, tuberculosis, cancer, etc.; ongoing steroid treatment and/or any kind of immunosuppressive therapy; currently participating or having participated in any other study three months before screening in the present study; past history of allergy to Chyawanprash-like products	• Chyawanprash Adult: 12 g bid. Children (5 to 12 years): 6 g bid. • Standard preventive measures/guidelines for COVID-19	• One cup of milk twice daily (approx. 200 mL) • Standard preventive measures/guidelines for COVID-19	90	In the intervention group and the control group, one and eight patients were COVID-19 positive, respectively	Dabur India Limited
Gupta et al., 2021 [17]	Prospective interventional parallel-arm randomized controlled trial. Allocated randomly 1:1 in either group using a computer-generated random numbers	CTRI/2020/05/025275	All healthcare workers (HCWs) aged between 25 and 60 years currently working in an environment with direct exposure to patients with confirmed COVID-19 infection	HCWs who declined consent, confirmed COVID-19 infection, already taking chloroquine/HCQ for any indication or any other prophylactic drug, pregnant or breastfeeding women, having a known comorbidity or an immuno-compromised state, and having a known allergy for the study drug	• Chyawanprash: 12 g bid • Standard of care	• Standard of care	30	No COVID-19 cases in either group during the study period, two months after completion of trial four patients of intervention and two subjects of control group developed COVID-19	NR
Gupta et al., 2021 [18]	A prospective, randomized, open-label, blinded end point, two-arm, comparative clinical study	CTRI/2020/09/027914	Patients aged 18 to 60 years with mild-to-moderate SARS-CoV-2 infection of not more than three days (≤3 days) as per the MoHFW Government of India (GoI) guidelines	COVID-19 infection, with immune-compromised status or any other medical or surgical condition that would require immediate medical or surgical intervention at the time of screening or may put the patient at increased risk during the study, AYUSH system-based contraindications, allergies or known to be allergic to intraperitoneal, pregnancy, lactation	Fixed Ayurvedic Regimen • Giloy Ki GhanVati (*Tinospora cordifolia*) tablet: 1 tablet, bid • Tulsi tablet (*Ocimum sanctum* Linn): 1 tablet, bid • Kalmegh (*Andrographis paniculata*) tablet: 1 tablet, bid • Chyawanprash: 10-12 g bid • Standard of care •Antibiotics/antiallergics/antipyretics/multivitamins as advised by local health authority.	• Conventional treatment/standard of care Antibiotics/antiallergics/antipyretics/multivitamins as prescribed/advised by local health authorities	28	Patients in the intervention group showed faster clinical recovery from the day of onset of symptoms by 51.34% (p < 0.05) as compared to the control group. A higher proportion of patients taking intervention recovered within the first two weeks compared to those taking only SOC. 5 times more patients recovered within seven days in the intervention group when compared to the control (p < 0.05) group	NR
Sanger et al., 2021 [19]	NR	NR	NR		• Chyawanprash SF • ICB03	• Standard of care	28	NR	NR

**Table 3:** Analytical description of included studies NR: not reported; AE: adverse events; SAE: serious adverse events.

First author, year	Intervention	Enrolled	Analyzed	Age (mean ± SD)	Male	COVID-19 status	HCRU for COVID-19 patients	Safety outcomes
Mata S, 2022 [16]	Ayuraksha Kit	141548	133782	38.4 ± 13.47	63785 (47.7%)	Patients who underwent RTPCR/RAT test: 5428; COVID-19 positive: 5367 (98.9%); COVID-19 patients who were symptomatic: 33/61 (54.9%)	Hospitalized: 6	AE: 1583 (0.01%)
	Standard preventive measures	19480	18514	38.2 ± 14.12	8387 (45.3%)	Patients who underwent RTPCR/RAT test: 422; COVID-19 positive: 414 (98.1%); COVID-19 patients who were symptomatic: 3/8 (37.5%)	Hospitalized: 1	NR
Godatwar PK, 2021 [10]	Chyawanprash	362	351	33.52 ± 12.46	194 (55.2%)	Patients who underwent RTPCR/RAT test: 42; COVID-19 positive: 1; symptomatic patients who underwent RTPCR/RAT test: 8; COVID-19 positive: 1	Hospitalized: 1	AE: 52 (14.8%); SAE: 1 (0.3%)
	One cup of milk twice daily; standard preventive measures/guidelines for COVID-19	359	345	31.73 ± 12.34	186 (53.9%)	Patients who underwent RTPCR/RAT test: 28; COVID-19 positive: 8; symptomatic patients who underwent RTPCR/RAT test: 11; COVID-19 positive: 8	Hospitalized: 4; ventilator support: 0; ICU admission: 1	AE: 54 (15.6%); SAE: 5 (1.4%)
Gupta A, 2021 [17]	Chyawanprash	100	98	32.122 ± 7.390	58 (59.2%)	During the study period COVID-19 positive: 0; COVID-19 positive at two-month follow-up: 2	Hospitalized: 0	No AE reported
	Standard of care	99	95	33.357 ± 8.567	47 (49.5%)	During the study period COVID-19 positive: 0; COVID-19 positive at two-month follow-up: 4	Hospitalized: 1	No AE reported
Gupta A, 2021 [18]	Fixed Ayurvedic regimen	35	35	39.20 ± 11.68	19 (54.2%)	NR	Oxygen support: 1; ICU admission: 1	AE: 4 (11.4%); SAE: 1 (2.8%)
	Conventional treatment/standard of care	33	33	41.06 ± 12.12	24 (72.7%)	NR	Oxygen support: 1	AE: 4 (12.1%)
Sanger NS, 2021 [19]	Chyawanprash SF, ICB03	60	60	NR	NR	NR	NR	In both the intervention groups 50% of subjects were relieved of cough, shortness of breath, and fatigue more effectively and in around 7 days
	Standard of care	30	30	NR	NR	NR	NR	NR

Meta-analysis was conducted in RevMan 5.4.1 (The Cochrane Collaboration, Copenhagen) to report forest plots, funnel plots, and risk of bias (ROB) assessment charts using the Cochrane ROB tool. We used a random effect model to determine the effect size for outcomes reported at least in two studies. As the outcomes reported were binary, the odds ratio was used to report the effect size [20]. Due to the limited number of studies included in the meta-analysis, sensitivity analysis was not conducted and the publication bias was determined visually through a funnel plot.

Result

Study Description

**Figure 1:**
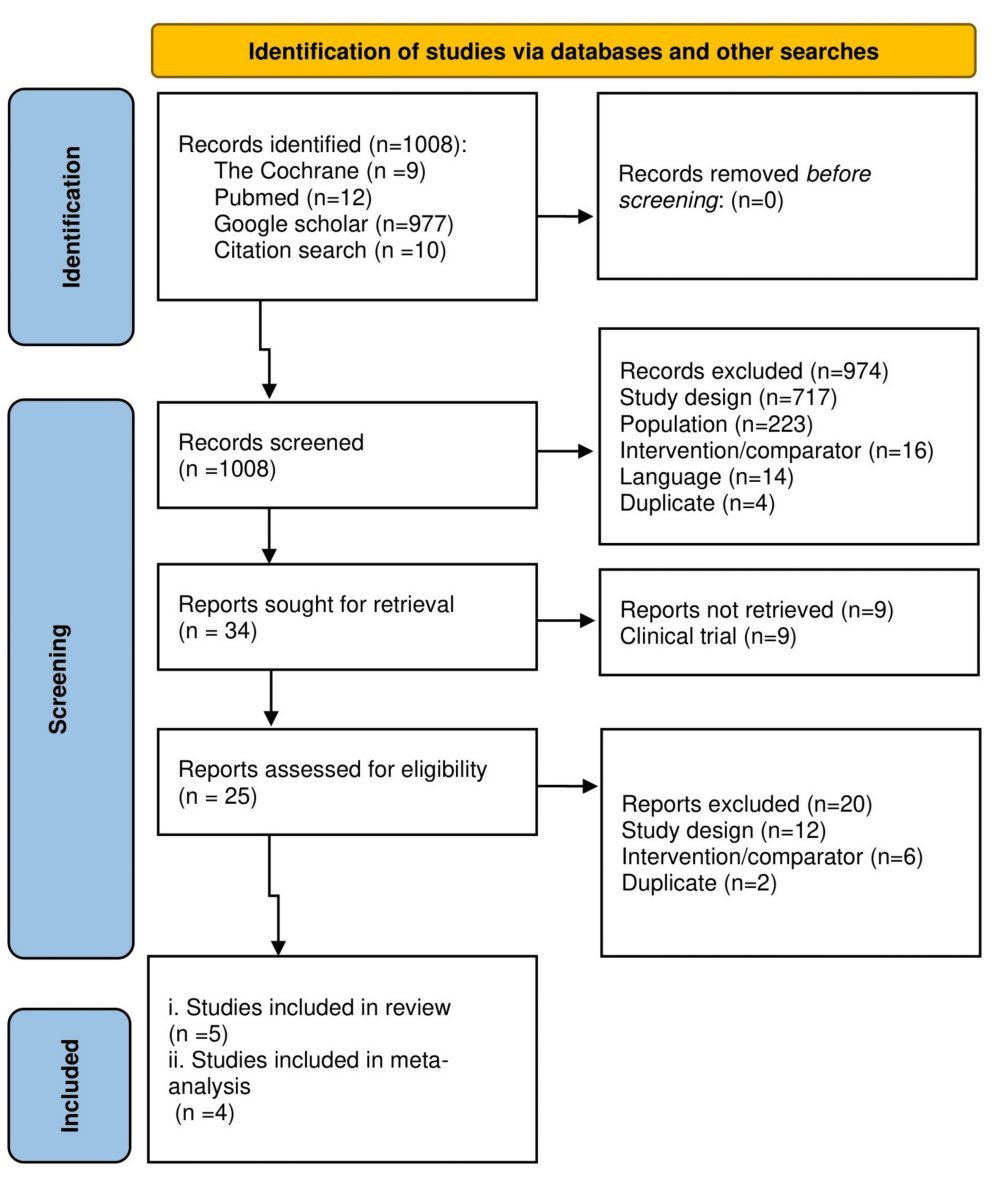
PRISMA chart

The search strategy resulted in 1008 articles from databases and through a citation search. Following title-abstract screening and excluding duplicates and non-relevant studies, 34 articles were selected for full-text retrieval. Due to the unavailability of relevant information, nine clinical trials were excluded; hence, 25 studies were fetched for level 2 screening. Finally, five studies were included in the systematic review (for one study only an abstract was available), and four studies were analyzed in the meta-analysis (Figure 1).

As per the PICOT, all relevant RCTs were included, which had a median (range) intervention duration of 30 (28 to 90) days [10,16-19] with one study reporting two-month follow-up details [17]. All studies were registered in the clinical trial registry India (CTRI) and were two-arm trials [10,16-18] except one study which was a three-arm trial with no CTRI registration detail available (only the abstract was fetchable) [19]. Mostly the trials were open-label RCTs and were conducted in India [10,16-19], with only two studies reporting their sponsor details [10,16]. The included studies enrolled 162,038 healthy individuals and 68 COVID-19 patients but analyzed 153,343 patients for whom the outcomes were reported. The intervention included Ayurvedic CP either as a standalone or in combination (with milk, fixed ayurvedic regime, etc.), whereas the comparator arm was provided with standard preventive measures or care for COVID-19 or milk [10,16-18] (Tables 2, 3). The ROB for the articles included in the meta-analysis is reported in Supplementary Table 5.

Outcomes

COVID-19 and Recovery Status

Four studies reported 5368 (96.4%) positive cases out of 5568 individuals who underwent intervention, whereas 422 (77.4%) positive cases were observed out of 545 individuals who were randomized in the control group. Different positivity rates were observed in symptomatic patients, 36 (50.7%) positive cases of 71 patients and 15 (65.2%) positive cases of 23 patients for participants randomized to intervention and comparator arms, respectively [10,16-18]. Only one study reported zero positive cases in both arms during the intervention period but at a two-month follow-up, intervention and comparator arms reported two and four positive cases, respectively [17]. In a between-group comparison, the intervention group has 12 times less incidence of COVID-19 infection (p < 0.05) compared to the control group. In symptomatic patients, the between-group comparison showed a significantly lesser (p < 0.05) number of subjects with COVID-19 positivity in the intervention compared to the control group [10].

Time taken for clinical recovery from COVID-19 based on average days (p < 0.001) and weeks (p < 0.05) was significantly less in the intervention group compared to the control group. Whereas the average number of days needed for the patients to turn COVID-19 negative was comparably less in the intervention arm than in the control arm but was non-significant (p > 0.05) [18].

HCRU and Severity

The HCRU details included days and number of COVID-19 patients hospitalized, oxygen support, and intensive care unit (ICU) utilization. Three studies reported outcomes for hospitalization: overall seven and six patients were hospitalized in the intervention and control arms, respectively [10,16,17]; the number of hospitalized days was four and the average days of hospitalization were 10.75, whereas another study reported an average (standard deviation) days of hospitalization as 7.80 (2.81) and 7.64 (3.17) in the intervention and control arms of both studies, respectively [10,18]. In the intervention arm, two patients required oxygen support and ICU admission, whereas in the control arm, only one patient needed oxygen support [18]. In another study, however, only one patient was admitted to ICU in the control arm [10]. In a study, six hospitalized patients were classified as mild (two patients) or moderate (two patients) in the intervention arm, whereas one patient in the control arm was classified as moderate [16]. According to the WHO ordinal scale [21], one patient in the intervention arm was graded 3 and afterward 0; however, eight patients in the control arm had been graded 3 and 2, respectively, at the beginning of the study, which changed to 0 (in seven patients) and 3 (in one patient) over the study period [10].

QoL and Patient-Reported Outcome (PRO)

The included studies used three scales to measure QoL: The visual analog scale (VAS) (appetite, bowel movement, and sleep were measured), the Quality of Life Enjoyment and Satisfaction questionnaire short form (Q-LES-Q-SF), and the WHO quality of life brief version (WHO QoL-BREF) (physical, psychological, social, and environmental health were measured) with all scales reporting 0 to 100 denoting poor to good effect, respectively. The PRO was reported as per the observation of physicians and patients based on a global assessment of efficacy and safety parameters. The QoL reported by VAS significantly improved in appetite, bowel, and sleep parameters in both the intervention (p < 0.001) group and control (p < 0.001) at 45 days compared to baseline, but the improvement was more profound in the intervention group [16]. Similarly, in the Gupta et al. trial, the WHO QoL-BREF reported physical, social, and environmental health improvement in the intervention group (p < 0.05) and non-significant improvement in the control group [18], whereas QoL assessed by Q-LES-Q-SF reported significant improvement only in the intervention group (p < 0.001), and significant deterioration in the control group (p < 0.001) at 90 days compared to baseline [10]. Similarly, Sanger et al. reported an overall improvement in QoL for both the intervention (Chyawanprash SF, ICB03), whereas no details were reported for the control group [19]. Moderately higher proportions of individuals randomized to the intervention group reported better PRO (very much improvement, good safety) compared to the control group based on the global assessment of efficacy and safety parameters [10,18].

Safety Outcomes

In both intervention and control groups, most adverse events or serious adverse events were infrequent and not life-threatening [16,18,19], with one study reporting no adverse events in either group [17]. According to physicians' and patients' evaluations of CP based on global safety assessment parameters, it has excellent overall safety [10]. The study reported low incidence rates of adverse effects, including nausea, vomiting, flatulence, constipation, and acidity/gastritis, all below 0.50% [16]. Both intervention and control groups significantly improved, with 50% of subjects experiencing relief from symptoms such as cough, shortness of breath, and fatigue within about seven days, along with reductions in biochemical parameters like CRP, IL-6, and TNF alpha levels [19]. Overall, the studies suggest that CP is safe with few adverse effects.

Biomarkers

Only two studies reported change in biomarkers such as hsCRP (high sensitive C-reactive protein), IL-6 (interleukin-6), IL-10 (interleukin-10), TNF alpha (tumor necrosis factor-alpha), IgG (immunoglobulin G), IgM (immunoglobulin M), and IgE (immunoglobulin E), with their levels remaining stable, indicating the safety of Ayurvedic interventions [16,17]. Among the studies, biomarker analysis post-intervention reported non-significant alteration in hsCRP, IL-6, IL-10, TNF alpha, IgG, IgM, and IgE values (the analysis was done for 18 patients only) [17].

Meta-analysis

**Figure 2:**
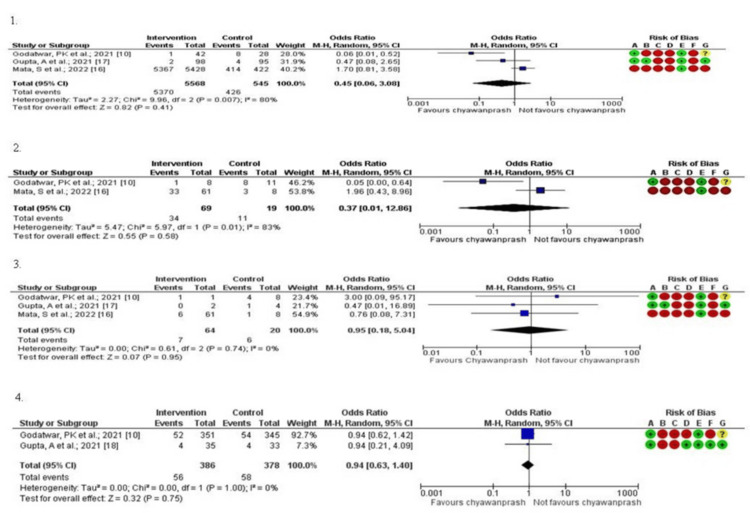
Forest plots 1. Forest plot for COVID-19-positive patients. 2. Forest plot for COVID-19 symptomatic patients. 3. Forest plot for HCRU (hospitalization). 4. Forest plot for adverse events. For ROB, A: Random sequence generation (selection bias); B: Allocation concealment (selection bias); C: Blinding of participants and personnel (performance bias); D: Blinding of outcome assessment (detection bias); E: Incomplete outcome data (attrition bias); F: Selective reporting (reporting bias); and G: Other bias. HCRU: healthcare resource utilization; ROB: risk of bias.

The meta-analysis incorporated four studies [10,16-18], each assessing the impact of CP on diverse health parameters. The odds ratios for COVID-19-positive patients, COVID-19-positive in symptomatic patients, HCRU (hospitalized), and adverse events were 0.45 (95% CI 0.06-3.08, p = 0.41), 0.37 (95% CI 0.01-12.86, p = 0.58), 0.95 (95% CI 0.18-5.04, p = 0.95), and 0.94 (95% CI 0.63-1.40, p = 0.75), respectively. High heterogeneity was observed for studies reporting COVID-19-positive patients (I^2^ = 80%, p = 0.007), COVID-19 positive in symptomatic patients (I^2 ^= 83%, p = 0.01), whereas HCRU (I^2^ = 0, p = 0.89) and adverse events (I^2^ = 0, p = 1.00) reported no heterogeneity but was not significant (Figure 2). In Supplementary Table 6, the values are reported which have been captured for meta-analysis.

Publication Bias

**Figure 3:**
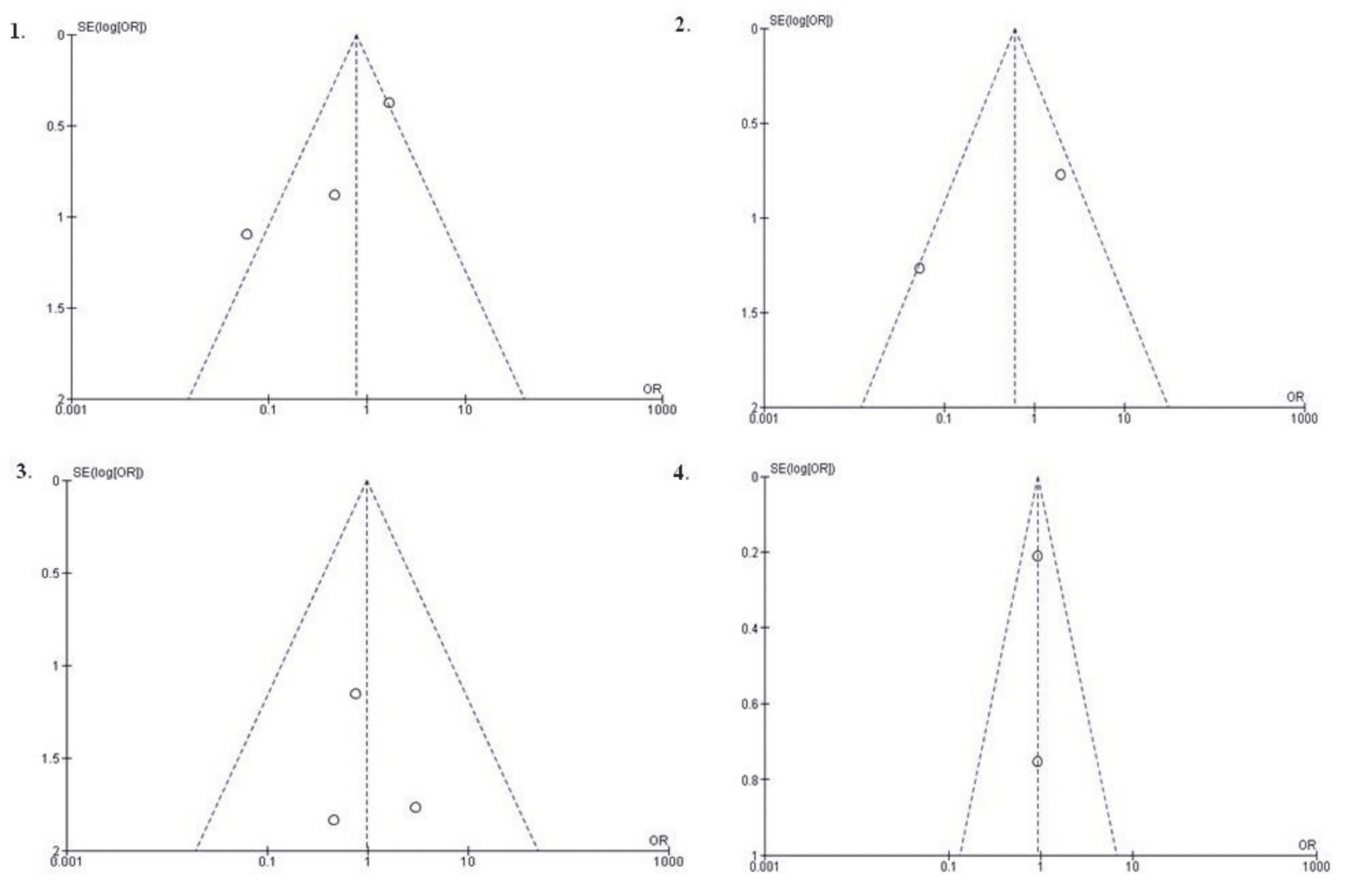
Funnel plots 1. Funnel plot for COVID-19-positive patients. 2. Funnel plot for COVID-19 symptomatic patients. 3. Funnel plot for HCRU (hospitalization). 4. Funnel plot for adverse events. HCRU: healthcare resource utilization; SE: standard error.

Publication bias was evaluated utilizing funnel plots. The majority of the studies were situated within the 95% confidence interval, as depicted visually, indicating a minimal likelihood of publication bias (Figure 3).

Discussion

This systematic review included five RCTs conducted in India reporting a total of 153,343 patients in the intervention (CP) and the control group with the median duration of the trial being 30 days. Overall the study reported that CP can have a positive impact on health by improving the QoL, HCRU, biomarkers, and better safety outcomes in symptomatic and asymptomatic COVID-19-positive patients. Although meta-analysis provided valuable insights into the efficacy and safety of CP in managing COVID-19, by reducing COVID-19 positivity, HCRU, and adverse events, the odds ratios were statistically insignificant. It could be ascribed to differences in the dosage of CP and manufacturing and brand differences that may have resulted in insignificant outcomes [22-24]. Even though CP is celebrated for its potential immunity and is consumed globally, the quantity and frequency of CP ingestion are not standardized by any clinical trials or real-world studies.

There were no meta-analyses demonstrating the safety or effectiveness of CP, hence a direct comparison was not feasible. Nevertheless, our outcomes were in tandem with a case series of five adult COVID-19-positive patients with varied symptoms, in which Ayurvedic intervention including CP reported complete recovery without any subsequent complications and hospitalizations [25]. Additionally, in a single-arm trial of healthcare workers exposed to COVID-19 infection (as part of their work), daily administration of 12 g of CP resulted in significantly fewer positive cases after the intervention than before [26]. In two clinical trials including healthy adults and children, consumption of CP was reported to result in better QoL, safety, and energy levels compared to the control arm [27,28]. As an additional immunity booster, the Indian Ministry of AYUSH has suggested consuming 10 g of CP in the morning [22].

Recent reviews and meta-analyses also suggest that combining AYUSH (Ayurveda, Yoga & Naturopathy, Unani, Siddha, and Homeopathy) prophylaxis with standard preventive measures may decrease the risk of COVID-19 infection by enhancing viral clearance and clinical recovery in mild to moderate cases, compared to standard of care [29-31].

Despite the lack of statistical significance, high heterogeneity, and publication bias (in one outcome), our findings may align with recent initiatives by the Government of India (GOI), particularly through the Ministry of AYUSH, which has recognized the potential of Ayurvedic interventions in combating COVID-19 [32-34]. This novel SRMA contributes evidence-based insights that shall facilitate healthcare practitioners, policymakers, and the general public to use CP in COVID-19 infection. With CP being a prominent component of Ayurvedic medicine, its inclusion in the armamentarium against COVID-19 holds promise.

## Conclusions

The systematic review of five RCTs highlighted CP's possible benefits in reducing positive cases, HCRU, and adverse events and improving QoL in COVID-19 patients. The meta-analysis outcomes may add weight to the growing body of evidence supporting the role of traditional medicines, especially CP, in complementing conventional approaches to COVID-19 management. However, it is crucial to acknowledge the need for further research to validate and contextualize these findings. Future studies should address the observed heterogeneity, employ standardized methodologies, and consider diverse patient populations.

## Appendices

**Table 4:** Search strategy with keywords and MeSH* * All searches in respective databases done after login.

S. No.	Database	MeSH/Keywords	Strategy	Hits
1	Cochrane (05/03/2023, 19:52:37)	Chyawanprash, chyavanprash, ratnaprash	Advanced search>Search manager> Jan 2020 to Jan 2023:	
			#1: Chyawanprash	14
			#2: Chyavanprash	3
			#3: Ratnaprash	0
			#4: #1 OR #2 OR #3	12
2	Google Scholar (18/03/2023)	Chyawanprash, chyavanprash, chyawanprash, chavanprash, ratnaprash, ratnaprasha	· Chyawanprash OR chyavanprash OR chawanprash OR chavanprash OR ratnaprash OR ratnaprasha · Filter (2020 to 2023)	977
3	PubMed (09/02/2023, 07:47:33)	Chawanprash, chyavanprash, ratnaprash	Advanced search:	
			#1: "Chawanprash"[All Fields]	1
			#2: "Chyavanprash"[Supplementary Concept] OR "Chyavanprash"[All Fields] OR "chyawanprash"[All Fields]	29
			#3: "Ratnaprash"[All Fields]	1
			#4: #1 OR #2 OR #3 AND (2020:2023[pdat])	9

**Table 5:** Risk-of-bias detail description

Study	Mata S, et al., 2022 [16]		Godatwar PK, et al., 2021 [10]		Gupta A, et al., 2021 [17]		Gupta A, et al., 2021 [18]	
	Risk	Reason	Risk	Reason	Risk	Reason	Risk	Reason
A. Random sequence generation (selection bias)	High	Cluster randomization	Low	Computer-generated randomization was followed	Low	Computer-generated randomization	Low	Computer-generated randomization chart was used
B. Allocation concealment (selection bias)	High	No allocation concealment was done	High	Allocation concealment was not followed	Low	The randomization code was given in opaque, sealed envelope	High	Open-label trial
C. Blinding of participants and personnel (performance bias)	High	Open label	High	Open-label trial, blinding might not have been followed	High	Open label	High	Open-label trial
D. Blinding of outcome assessment (detection bias)	High	Open label	High	Open-label trial, blinding might not have been followed	High	Open-label trial, blinding might not have been followed	Low	Blinded end point assessment
E. Incomplete outcome data (attrition bias)	Low	Low attrition (<10%)	Low	The attrition was small and similar across groups	Low	The attrition level was low (<10%)	Low	All patients were analyzed with no attrition
F. Selective reporting (reporting bias)	High	Certain outcomes not reported for control group	High	Outcomes for all patients who received treatment were not analyzed	High	Some outcomes were not evaluated in all patients	Low	Outcomes were reported for all patients underwent randomization
G. Other bias	High	The sample was less than what was calculated	Unclear	The study was conducted in the region where incidence of COVID-19 was high	Low	Statistical analysis was conducted for sample size	Low	Statistical analysis was conducted for sample size

**Table 6:** Data points for meta-analysis NR: not reported. *This data was not considered in the meta-analysis.

S. No.	Study ID	Intervention	COVID-19 tested	COVID-19 positive	COVID-19 tested who were symptomatic	COVID-19 positive who were symptomatic	HCRU in COVID-19 positive (n/N)	Adverse events (n/N)
1	Mata S, et al., 2022 [16]	- Ayuraksha Kit Chyawanprasha - Standard preventive measures/guidelines for COVID-19	5428	5367	61	33	6/61	NR
		- Standard preventive measures/guidelines for COVID-19	422	414	8	3	1/8	NR
2	Godatwar PK, et al., 2021 [10]	- Chyawanprash - Standard preventive measures/guidelines for COVID-19	42	1	8	1	1/1	52/351
		- One cup of milk twice daily- Standard preventive measures/guidelines for COVID-19	28	8	11	8	4/8	54/345
3	Gupta A, et al., 2021 [17]	- Chyawanprash - Standard of care	98	2	NR	NR	0/2	0/98*
		- Standard of care	95	4	NR	NR	1/4	0/95*
4	Gupta A, et al., 2021 [18]	- Fixed Ayurvedic regimen - Conventional treatment/standard of care	NR	NR	NR	NR	NR	4/35
		- Conventional treatment/standard of care	NR	NR	NR	NR	NR	4/33
